# Prevalence and viral load determination of BK polyomavirus among Iranian patients with brain tumors

**DOI:** 10.22088/cjim.12.2.173

**Published:** 2021-03

**Authors:** Saghar Saber Amoli, Arghavan Zebardast, Hossein Keyvani, Yousef Yahyapour, Seyed Mohammad Ghodsi, Mahmood Maniati, Farzin Sadeghi

**Affiliations:** 1 Department of Microbiology, School of Medicine, Babol University of Medical Sciences, Babol, Iran; 2 Department of Virology, School of Public Health, Tehran University of Medical Sciences, Tehran, Iran; 3 Department of Virology, School of Medicine, Iran University of Medical Sciences, Tehran, Iran; 4 Infectious Diseases & Tropical Medicine Research Center, Health Research Institute, Babol University of Medical Sciences, Babol, Iran; 5 Department of Neurosurgery, Shariati Hospital, Tehran University of Medical Sciences, Tehran, Iran; 6 School of Medicine, Ahvaz Jundishapur University of Medical Sciences, Ahvaz, Iran; 7 Cellular & Molecular Biology Research Center, Health Research Institute, Babol University of Medical Sciences, Babol, Iran

**Keywords:** BK human polyomavirus, Brain tumors, Tumor antigen

## Abstract

**Background:**

Due to persistent infections of human central nervous system (CNS), polyomaviruses have been identified as one of the risk factors for brain tumor development. Human BK virus is of signiﬁcant interest due to its experimental neuro-oncogenic potential and the possible association with CNS neoplasms. However, the results of different studies are discrepant. In the present study, we aimed to investigate the prevalence of BK virus genome and quantify BK viral load in Iranian patients with primary and metastatic brain malignancies.

**Methods:**

To assess the prevalence of BK virus sequences, a total of 58 fresh brain tumors were examined by quantitative real-time PCR. The BK viral load was determined as viral copy number per cell.

**Results:**

Of the 58 brain tumor samples BK tumor antigen (TAg) sequences were detected in 26 (44.8%) of cases. In primary brain tumors, BK virus sequences were recognized more frequently in schwannomas (15.5%) and meningiomas (12.1%). The mean BK virus TAg copy number in positive cases was 0.20×10^-3^±0.27×10^-3^ (range 0.01×10^-3^- 0.8×10^-3^) copies per cell.

**Conclusion:**

Taken together, in the present study low copy numbers of BK virus TAg gene was detected in brain tumor cells, which can indicate that BK virus may contribute to tumor induction by indirect mechanisms or neuro-persistence of this virus without any pathological consequences.

Central nervous system (CNS) tumors are diverse groups of neoplasms originating from the brain, spinal cord or meninges and accounting for 2% of all adulthood malignancies (1). Brain tumors comprise a diverse group of tumors, accounting for 1.3% of all adulthood cancers and 17% of childhood malignancies (1). The etiological cause of CNS tumors is not well understood, and little proven risk factors have been identified to date. Although some genetic diseases, gene polymorphisms and exposure to ionizing radiation have been identified as a potential risk factors for brain tumors (2), other risk factors, especially viral infections, may also play a role in tumor formation (3). Among viral agents, polyomaviruses are more important due to the persistent infection in the CNS, ability to transform neural cells in culture and induction of brain tumors in animal models (4). Inoculation of human polyomaviruses into hamster induces different types of brain tumors. 

In addition, intracerebral, subcutaneous and intravenous inoculation of these viruses into new world monkeys’ results in astrocytoma, glioblastoma and neuroblastoma (5, 6). Polyomaviruses are non-enveloped double stranded DNA viruses with icosahedral symmetry and a size ranging from 45 to 50 nm. The family comprises 14 human polyomaviruses (7). The most prominent of which are JC, BK and Merkel cell polyomavirus (MCPyV). Initial infection with human polyomaviruses usually occurs at an early age and does not lead to acute disease (8). 

Low levels of polyomaviruses are maintained in individuals with competent immune system, but following immunodeficiency or immunosuppression, these viruses replicate at very high levels and lead to disease. Due to having tumor antigen (TAg) oncogenes, human polyomaviruses can interact with tumor suppressor proteins including retinoblastoma protein (pRb) and p53 and offer a possible mechanism of oncogenicity. However, current evidence only supports the role of MCPyV as a human carcinogen, lead to Merkel cell carcinoma (MCC) (8). 

In MCCs, parts of the virus genome, in particular TAg oncogene sequences; have been integrated into the host cell DNA (9). The first evidence of the possible association of BK virus with human CNS tumors was obtained by two separate studies in 1987. Coralini et al. and Doriso et al. identified the BK virus genome in 25% and 46% of human brain tumors, respectively (10, 11).

Since that reports, there have been numerous studies on the role of BK virus in the development of human CNS tumors. However, the results of various studies are inconsistent and the role of BK virus in CNS tumor development is still highly controversial (12, 13). Therefore, further studies on the role of BK virus in the development of CNS tumors are necessary. The aim of present study was to evaluate the genome prevalence and to determine the copy number of BK virus TAg sequences in patients with primary and metastatic brain malignancies.

## Methods

**Patients and Samples:** The present study was performed on 58 patients with brain tumor referred to 2 neurosurgical reference Hospitals of Tehran, Shariati Hospital and Khatam Al Anbia Hospital, from November 2012 to January 2014. After obtaining written informed consent, fresh biopsy specimen of brain tumor were surgically removed and immersed in RNALater solution (Sigma R0901, St. Louis, MO, USA). Fresh biopsy specimens were immediately transferred to the study site on the same day of surgery and stored in a -70° C freezer. Patients with primary and metastatic brain tumor were included in the study. Patients with positive test for human immunodeficiency virus type 1 (HIV-1) antigen or antibody and subjects who received immunosuppressive therapy were excluded from investigation. This study was approved by the Ethics Committee of Tehran University of Medical Sciences (Project code: 92-01-30-20990) and informed consent was obtained from all participants.

**DNA Extraction:** The QIAamp DNA mini kit (QIAGEN GmbH, Hilden Germany) was used to extract DNA from the tissue samples based on the manufacturer’s instructions. NanoDrop spectrophotometer was used to estimate the concentration and quality of the extracted DNA (Thermo Scientific Wilmington, USA). Concentrations of DNA extracted from tissue were in the range of 450 - 500 ng/μl and their A260/A280 ratio was in the range of 1.7 to 1.9 indicating optimum concentration and purity.

**BK virus Quantitative Real-Time PCR: **A TaqMan real-time PCR method was used to assess the presence of BK virus TAg oncogene and to determine the amount of viral load using the Rotor-Gene® Q Real-time PCR System (QIAGEN GmbH, Hilden Germany) according to a previously described procedure (14). A proven single-copy cellular gene (human RNase-P gene) was utilized to normalize viral copies to the number of cell equivalents as described previously (15). Each real-time PCR reaction consisted of 500 ng of purified DNA, 12.5 µl Maxima Probe qPCR Master Mix (Fermentas, Glen Burnie, MD), 0.3 mM of each primer, and 0.2 mM of TaqMan probe in a 25 µl total reaction volume.

**Figure. 1:**
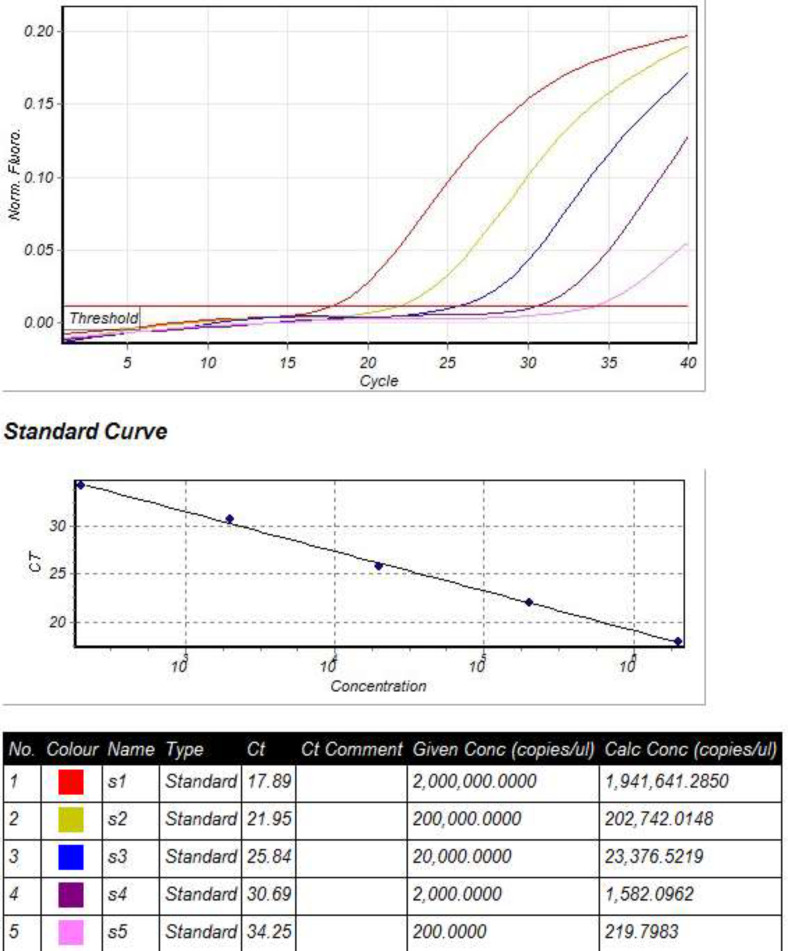
Results of Real Time PCR test on different dilutions of standard sample (recombinant plasmid containing TAg region of BK virus) and standard curve

**Standard curve for quantitative real-time PCR:** To construct quantitative standards for real-time PCR, the BK virus genome was extracted from urine samples of an HIV positive patient. After DNA extraction, TAg region of the BK virus genome was amplified by conventional PCR (16), and then cloned into the pTZ57R / T PCR cloning vector (InsTAclone^TM^ PCR Cloning Kit, Fermentas, MD, USA). The recombinant plasmids were extracted from the grown colonies and then confirmed by sequencing. To determine the copy number of the human RNase P gene, a conserved region of this gene was amplified using specific primers (17) and cloned into the pTZ57R / T vector. The recombinant plasmids containing the TAg region of BK virus and human RNase P gene were used as quantitative standards for real-time PCR and BK viral load as a copy number/cell was determined according to a previously described procedure (16) (figure 1).

**Statistical Analysis:** The statistical analysis was done using SPSS Version 16 software (SPSS Inc., Chicago, IL, USA). The χ2-test was used to evaluate the associations between categorical variables. Normal distribution of the variables was analyzed by Kolmogorov–Smirnov test. Analysis of continuous variables was performed using the independent-samples t-test /Mann–Whitney U test and ANOVA / Kruskal-Wallis test. A p-value of ≤0.05 was considered to be statistically significant.

## Results

This study included 58 brain tumor samples. Regarding brain tumor origin, 54 (93.1%) were primary and four (6.9%) metastatic brain tumors. Demographic, clinical and histopathologic parameters of BK positive and negative patients are presented in table 1. In the present study, BK virus TAg sequence was detected in 44.8% (26 out 58) of brain tumor samples. In detail, among the primary and positive brain tumors for BK virus TAg sequence, schwannomas (15.5%) and meningiomas (8.2%) were more common (table 2). According to table 2, there was no significant relationship between BK virus TAg positivity and type of brain tumor (P=0.575). Out of four metastatic brain tumors, three were diagnosed as adenocarcinoma originating from lung cancer and one was diagnosed to be squamous-cell carcinoma with an unknown primary site of origin. The BK virus TAg sequence was detected in 2 out of 3 adenocarcinoma specimens. The only squamous-cell carcinoma metastatic brain tumor was negative for BK virus

**Table.1:** Demographic, clinical and histopathologic parameters of the studied patients

**Patients**		**BK T-Ag Sequence**			
		**Positive**	**Negative**	**Total**	**P-value**
No of patients		26 (44.8%)	32 (55.1%)	58	
Age (years)		49.5±15.7	43.1±18.7	46±18.6	0.17
Sex	Male Female	13 (22.4%)	15 (25.8%)	28 (48.2%)	0.813
		13 (22.4%)	17 (29.3%)	30 (51.7%)	
WBC ^a^ Counts per microliter		8402.6±2760.3	8468.2±2592.7	8438.8±2645.5	0.833
Histopathology	Schwannoma	9 (64.3%)	5 (35.7%)	14 (24.1%)	0.575
	Meningioma	7 (58.3%)	5 (41.7%)	12 (20.7%)	
	Glioblastoma multiform	2 (28.6%)	5 (71.4%)	7 (12.1%)	
	Astrocytoma	1 (33.3%)	2 (66.7%)	3 (5.2%)	
	Pituitary adenoma	1 (33.3%)	2 (66.7%)	3 (5.2%)	
	Epidermoid Tumor	0 (0%)	3 (100%)	3 (5.2%)	
	Adenocarcinoma(Metastatic)	2 (66.7%)	1 (33.3%)	3 (5.2%)	
	Hemangioblastoma	1 (50%)	1 (50%)	2 (3.4%)	
	Pineoblastoma	0 (0%)	2 (100%)	2 (3.4%)	
	Oligodendroglioma	1 (50%)	1 (50%)	2 (3.4%)	
	Oligoastrocytoma	0 (0%)	1 (100%)	1 (1.7%)	
	Chordoma	1(100%)	0 (0%)	1 (1.7%)	
	Squamous cell carcinoma(Metastatic)	0 (0%)	1 (100%)	1 (1.7%)	
	Cavernoma	1 (100%)	0 (0%)	1 (1.7%)	
	Medulloblastoma	0 (0%)	1 (100%)	1 (1.7%)	
	Xanthoastrocytoma	0 (0%)	1 (100%)	1 (1.7%)	
	Ependymoma	0 (0%)	1 (100%)	1 (1.7%)	

* Continuous parameters are presented as mean ± standard deviation

^a ^White blood cell

**Figure. 2:**
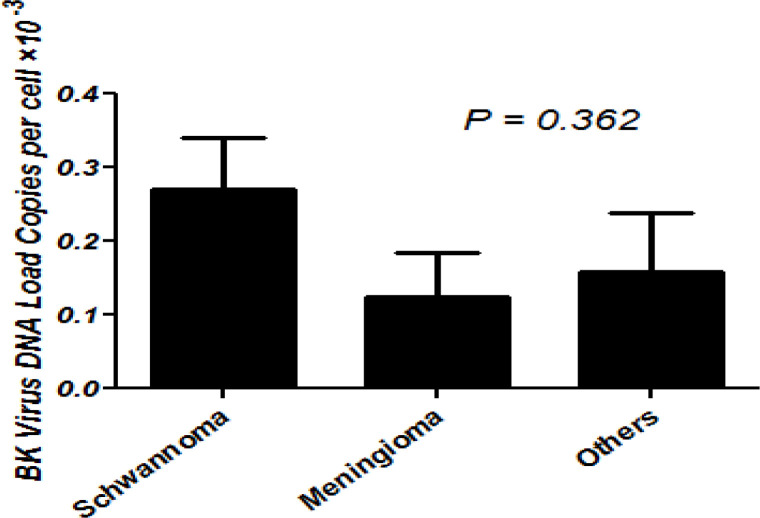
The mean BK virus TAg DNA load in BK-positive brain Tumors. The group of others includes BK-positive cases with glioblastoma multiform, astrocytoma, pituitary adenoma, hemangioblastoma, oligodendroglioma, chordoma, cavernoma and metastatic adenocarcinoma. Error bars indicate standard error. The P-value was determined by the Kruskal-Wallis test

In the present study, tissue samples from the different types of brain tumors were evaluated by quantitative real time PCR for the BK virus TAg gene in each tumor cell. In brain tumor positive samples, the mean BK virus TAg gene copy number per tumor cell was 0.20×10^-3 ^±0.27×10^-3 ^(0.01×10^-3^ – 0.8. 10^-3-^copies per cell). In the current investigation, due to lower frequency of other tumors compared with schwannoma and meningioma we placed them in a new group called others for statistical analysis. The mean BK virus TAg gene copy number was higher in schwannoma tumors compared with others and meningioma groups (figure 2). However, there was no significant difference between the mean copy number of BK virus TAg in the tumors of schwannoma compared with others and meningioma groups (P=0.362).

**Table.2:** Summary of studies that have identified BK virus TAg DNA Sequences, Oncoprotein in various types of brain tumors

**Pathological diagnosis and histology of the tumor**	**BK virus TAg Oncoprotein (%)**	**BK virus TAg DNA Sequences (%)**	**Pathological Diagnosis of Tumor**
(10) Corallini et al	5	25	Astrosytoma
(19) Martini et al	100	94	
(24) Delbue et al	-	20	
(9) Corallini et al	-	17	Glioblastoma multiform
(11) Dorries et al	10	35	
(13) Rollison et al	-	3	
(24) Delbue et al	-	9.5	
(9) Corallini et al	20	65	Ependymoma
(23) Negrini et al	-	60	
(19) Martini et al	-	91	
(10) Corallini et al	-	13	Oligodendroglioma
(19) Martini et a	-	78	
(11) Dorries et al	-	83	Meningioma
(19) Martini et al	-	57	
(10) Corallini et al	-	10	Schwannoma
(11) Dorries et al	-	5	

## Discussion

In the present study, which was performed on 58 patients with brain tumor, schwannoma (24.1%), meningioma (20.7%) and glioblastoma multiform (12.1%) were the most common types of tumors. The prevalence pattern observed in this study is consistent with the reports of brain tumor prevalence from the developing countries, and with the results of the most recent systematic review of 10802 brain tumor cases in Iran (18). In the current investigation, low copy number of TAg gene of BK virus was detected in 44.8% of brain tumor specimens. Schwannomas and meningiomas were the most common primary tumors positive for BK virus TAg gene. In addition, from 4 metastatic brain tumor specimens in this study, BK virus TAg gene was identified in 2 cases of adenocarcinoma (metastasis from lung tissue) with a mean copy number of 0.3× 10^-3^±0.27×10^-3^copies per cell. The first evidence of the possible association of BK virus with brain tumors was obtained by two separate studies in 1987. Corallino et al. using southern blot technique, identified the BK virus genome in 25% of their brain tumors (10), then Doris et al. identified the BK virus genome in 46% of the brain tumors (11). Analysis for BK virus DNA conducted by Doris et al. revealed the presence of BK virus-specific sequences in 46% of brain tumor specimens which was in agreement with our findings. It should be noted that Doris et al. also examined 29 individuals without CNS tumors and no BK virus was found in these subjects. 

In a study conducted by Martin et al., BK virus genome was identified in 57% of meningioma tumors; these findings might be relevant with our results in terms of BK virus prevalence rate in meningiomas (19). In this study, two glioblastoma tissue tested positive for BK virus. Interestingly a study by Rollison et al. (13) obtained similar results after analyzing the same series of tissues in 2 independent laboratories. In addition, Delbue et al. (24) demonstrated BK virus TAg sequences in meningioma and glioblastoma tumor which was consistent with our results. Moreover, DNA and oncoprotein of BK virus was identified by Flagstadt et al. in a substantial portion of neuroblastoma tumors. The interaction of TAg with p53 protein was confirmed in a number of samples of this study (20). Also, episomal BK virus DNA was detected in primary human brain tumors and cell lines from brain tumors by Negrini et al. (23). The studies that have managed to identify BK virus DNA or TAg oncoprotein in different types of brain tumor are summarized in table 5. Some studies have also rejected the existence of the BK virus genome or oncoprotein in brain tumors, including Arthur et al. and Kim et al. (21, 22) that are inconsistent with our results. Considering the role of BK virus in the stimulation of tumor cell migration and metastasis, to our knowledge, there is no study on brain tumor, however, a report revealed metastatic bladder carcinoma was causally associated to BK virus (25).

In a positive agreement with a previous study, current investigation revealed a low copy number of BK virus TAg gene per cell in primary and metastatic brain tumors (10). In addition, our finding was consistent with the results of previous reports, which have detected low copies of BK virus genome in human malignant cell lines and animal models (26, 27). A number of limitations exist in the current study that should be noted. First and foremost, access to normal and non-cancerous brain samples as a control group was impossible. Despite the fact that sampling from non-tumor and normal brain samples is morally unacceptable, future studies can examine autopsy brain tissue of individuals who died for non-brain tumor reasons. In addition, evaluation of the interaction between viral TAg and tumor suppressor proteins in tumor tissue using immunohistochemical techniques can provide valuable information regarding BK virus carcinogenesis. Investigating the infection status of patients and determining the number of copies of the virus in other locations, such as blood and urine, may also be useful in analyzing the information on the presence of the virus in the brain tumor, which is recommended for future studies.

Taken together, in the present study low copy numbers of BK virus TAg gene was detected in tumor cells, which can indicate that BK virus may contribute to tumor induction by indirect mechanisms or neuro-persistence of this virus without any pathological consequences. These results should urge further worldwide epidemiological and virological studies to distinguish the possible role of BK virus in tumor formation from simple persistent viral replication.

## References

[1] Siegel R, Naishadham D, Jemal A (2013). Cancer statistics, 2013. CA: Cancer J Clin.

[2] Preston-Martin S (1996). Epidemiology of primary CNS neoplasms. Neurol Clin.

[3] Saddawi-Konefka R, Crawford JR (2010). Chronic viral infection and primary central nervous system malignancy. J Neuroimmune Pharmacol.

[4] Croul S, Otte J, Khalili K (2003). Brain tumors and polyomaviruses. J Neurovirol.

[5] White MK, Gordon J, Reiss K (2005). Human polyomaviruses and brain tumors. Brain Res Rev.

[6] Miller NR, McKeever P, London W (1984). Brain tumors of owl monkeys inoculated with JC virus contain the JC virus genome. J Virol.

[7] Tahseen D, Rady PL, Tyring SK (2020). Human polyomavirus modulation of the host DNA damage response. Virus Genes.

[8] Gjoerup O, Chang Y (2010). Update on human polyomaviruses and cancer. Adv Cancer Res.

[9] Shuda M, Feng H, Kwun HJ (2008). T antigen mutations are a human tumor-specific signature for Merkel cell polyomavirus. Proc Natl Acad Sci USA.

[10] Corallini A, Pagnani M, Viadana P (1987). Association of BK virus with human brain tumors and tumors of pancreatic islets. Int J Cancer.

[11] Dörries K, Loeber G, Meixensberger J (1987). Association of polyomaviruses JC, SV40, and BK with human brain tumors. Virology.

[12] Abend JR, Jiang M, Imperiale MJ (2009). BK virus and human cancer: innocent until proven guilty. Semin Cancer Biol.

[13] Rollison DE, Utaipat U, Ryschkewitsch C (2005). Investigation of human brain tumors for the presence of polyomavirus genome sequences by two independent laboratories. Int J Cancer.

[14] MacKenzie J, Wilson KS, Perry J, Gallagher A, Jarrett RF (2003). Association between simian virus 40 DNA and lymphoma in the United Kingdom. J Nat Cancer Inst.

[15] Imajoh M, Hashida Y, Taniguchi A, Kamioka M, Daibata M (2012). Novel human polyomaviruses, Merkel cell polyomavirus and human polyomavirus 9, in Japanese chronic lymphocytic leukemia cases. J Hematol Oncol.

[16] McNees AL, White ZS, Zanwar P, Vilchez RA, Butel JS (2005). Specific and quantitative detection of human polyomaviruses BKV, JCV, and SV40 by real time PCR. J Clin Virol.

[17] Sadeghi F, Salehi-Vaziri M, Ghodsi SM (2015). Prevalence of JC polyomavirus large T antigen sequences among Iranian patients with central nervous system tumors. Arch Virol.

[18] Jazayeri SB, Rahimi-Movaghar V, Shokraneh F, Saadat S, Ramezani R (2013). Epidemiology of primary CNS tumors in Iran: a systematic. Asian Pac J Cancer Prev.

[19] Martini F, Iaccheri L, Lazzarin L (1996). SV40 early region and large T antigen in human brain tumors, peripheral blood cells, and sperm fluids from healthy individuals. Cancer Res.

[20] Flægstad T, Andresen PA, Johnsen JI (1999). A possible contributory role of BK virus infection in neuroblastoma development. Cancer Res.

[21] Arthur RR, Grossman SA, Ronnett BM (1994). Lack of association of human polyomaviruses with human brain tumors. J Neuro Oncol.

[22] Kim JY, Koralnik IJ, LeFave M (2002). Medulloblastomas and primitive neuroectodermal tumors rarely contain polyomavirus DNA sequences. Neuro Oncol.

[23] Negrini M, Rimessi P, Mantovani C (1990). Characterization of BK virus variants rescued from human tumours and tumour cell lines. J Gen Virol.

[24] Delbue S, Pagani E, Guerini FR (2005). Distribution, characterization and significance of polyomavirus genomic sequences in tumors of the brain and its covering. J Med Virol.

[25] Geetha D, Tong BC, Racusen L, Markowitz JS, Westra WH (2002). Bladder carcinoma in a transplant recipient: evidence to implicate the BK human polyomavirus as a causal transforming agent. Transplantation.

[26] Wold WS, Mackey JK, Brackmann KH (1978). Analysis of human tumors and human malignant cell lines for BK virus-specific DNA sequences. Proc Natl Acad Sci U S A.

[27] Pater MM, Pater A, di Mayorca G, Beth E, Giraldo G (1982). BK virus-transformed inbred hamster brain cells: status of viral DNA in subclones. Mol Cell Biol.

